# Electroacupuncture Ameliorates Postoperative Cognitive Dysfunction and Oxidative Stress via the SIRT1/FOXO1 Autophagy Pathway: An Animal Study

**DOI:** 10.1111/jcmm.70436

**Published:** 2025-02-26

**Authors:** Xiaojia Zhang, Binsen Zhang, Xiaoyu Qin, Lu Tang, Chunai Wang

**Affiliations:** ^1^ The First Clinical Medical College Gansu University of Chinese Medicine Lanzhou Gansu China; ^2^ Anesthesia and Pain Medical Center Gansu Hospital of Traditional Chinese Medicine Lanzhou Gansu China

**Keywords:** autophagy, cognitive dysfunction, electroacupuncture, oxidative stress, postoperative cognitive complications, postoperative cognitive dysfunction, SIRT1/FOXO1 signalling pathway

## Abstract

Postoperative cognitive dysfunction (POCD) is common in older adult patients and affects their prognosis. Studies suggested that autophagy and oxidative stress are key factors in the pathogenesis of POCD. This study aimed to determine whether electroacupuncture (EA) pre‐treatment improves POCD in aged rats and the underlying mechanisms. We established a model of POCD by using propofol anaesthesia and caesarean section in aged mice and assessed whether electroacupuncture at the Baihui and Neiguan points modulates autophagy and oxidative stress involved in the pathological process of POCD. The Morris water maze test assessed postoperative cognitive function. Oxidative stress was assessed using flow cytometry and enzyme‐linked immunosorbent assay (ELISA) to determine the levels of superoxide dismutase (SOD), reactive oxygen species (ROS) and malondialdehyde (MDA). Transmission electron microscopy was used to observe the ultrastructure of the hippocampal cord neurons. In addition, protein blotting and quantitative real‐time polymerase chain reaction (PCR) assays were performed to assess SIRT1, FOXO1, and autophagy markers at both the protein and mRNA levels. The results showed that anaesthesia/surgery significantly impaired cognitive performance, increased oxidative stress, decreased autophagy in the hippocampus, damaged hippocampal neurones and disrupted the mitochondrial structure in aged rats. EA pre‐treatment improved cognitive function, restored neuronal and mitochondrial function, increased Beclin‐1 and SIRT1 levels and attenuated oxidative damage and autophagy dysfunction in POCD rats. In conclusion, EA pre‐treatment improved POCD in aged rats, and this mechanism may be related to the enhancement of autophagy and the inhibition of oxidative stress through SIRT1/FOXO1 signalling.

## 1 Introduction

Postoperative cognitive dysfunction (POCD) is a neurological complication that frequently occurs in older adult patients. The main clinical manifestations of POCD include reduced attention, memory, cognition, and executive dysfunction [1, 2]. POCD can last for several months, which seriously affects the prognosis and daily life of patients and increases the cost of perioperative care [3, 4, 5]. Given the large number of surgical procedures performed on the older adult population, the prevention and treatment of POCD have become a global concern. The pathogenesis of POCD is complex and varied. It has been shown that POCD is closely related to postoperative inflammation [6], neuronal dysfunction and mitochondrial function, and that oxidative stress and autophagy play a key role in the development of POCD. Despite numerous clinical studies on the pathogenesis and prevention of POCD, its aetiopathogenesis remains unclear.

Autophagy is a cellular pathway involved in the degradation of organelles and proteins in the body [7]. Autophagy is categorised into three types according to how it delivers substances to lysosomes: macroautophagy, microautophagy and chaperone‐mediated autophagy [8]. Autophagy is involved in maintaining normal cellular and body functions [9]. Disruption of autophagic homeostasis can activate apoptosis or lead to the aggregation of many abnormally folded proteins and damaged organelles in the cell; the pathogenic microorganisms that invade the cytoplasm are not cleared promptly, which leads to the disruption of cellular homeostasis and cell death. Autophagy is involved in the pathogenesis of neurodegenerative diseases [10, 11], and mitochondrial autophagy is usually responsible for the pathological deterioration of Alzheimer's disease [12]. Studies have shown that autophagy can ameliorate Alzheimer's disease by degrading extracellular Aβ plaques [13]. Increasing evidence suggests that dysregulation of autophagy may contribute to the development of POCD. Increased autophagy has been reported to improve cognitive function and alleviate postoperative cognitive impairment by regulating lipid metabolism and inflammation [14].

The silencing information factor sirt1 and SIRT1 (Sir2) are NAD‐dependent deacetylases involved in regulating metabolism, immune responses, and ageing [15, 16]. Studies have shown that sirt1 inhibits pro‐inflammatory cytokines and hippocampal microglial activation, regulates autophagy and oxidative stress and improves postoperative cognitive function in aged rats [17, 18, 19]. SIRT1 is a conserved NAD + ‐dependent deacetylase that plays a key role in many important life activities, including metabolism, immune responses, brain development and ageing [16, 20]. SIRT1 is widely expressed in the central nervous system, and it is mainly distributed in brain functional areas such as the hippocampus and prefrontal cortex, as well as in the hypothalamus and the cerebellum [21]. SIRT1 is significantly downregulated in neurodegenerative diseases, acute kidney injury, cancer, and many other diseases [22, 23, 24]. FOXO1, a member of the forkhead box (FOXO) family, is a key regulator of a wide range of physiological processes, including the regulation of apoptosis, autophagy, antioxidant enzymes, cell cycle arrest genes, metabolism, and immunity modulation [25, 26, 27]. The Sirt1/FOXO1 signalling pathway is involved in the regulation of various physiological processes. Studies have shown that SIRT1 promotes the transfer of FOXO1 from the nucleus to the cytoplasm through deacetylation. When SIRT1 is activated, downstream FOXO1 and PGC‐1α are deacetylated, exerting anti‐oxidative stress, anti‐apoptotic, and anti‐inflammatory effects [28]. The SIRT1/FOXO1 signalling pathway regulates a wide range of important life activities, including metabolism, brain development, ageing, immune responses, and regulating neuroinflammation in various tissues [15, 19].

Electroacupuncture (EA) is a commonly used therapeutic tool in Chinese medicine that uses an EA stimulator to pass a continuous and stable electric current through acupuncture needles, choose different waveforms (e.g., continuous or intermittent) and stimulate acupuncture points to play a therapeutic role according to the traditional Chinese meridian theory [29]. EA stimulation can inhibit neuroinflammation, reduce oxidative stress, inhibit neuronal apoptosis and synaptic degeneration and improve neurocognition and has been widely used in treating various neurodegenerative disorders [30]. Several studies have shown that EA stimulation improves postoperative cognition and exerts neuroprotective effects [31, 32, 33]. However, the therapeutic mechanisms underlying the Sirt1/FOXO1 pathway in POCD remain unclear. Therefore, this study aimed to investigate whether the mechanism by which EA pre‐treatment improves POCD in aged rats is related to the upregulation of autophagy and inhibition of oxidative stress via the Sirt1/FOXO1 pathway. We hypothesised that EA pre‐treatment activates autophagy and inhibits oxidative stress by activating the Sirt1/FOXO1 pathway, thereby improving postoperative cognitive function.

## 2 Materials and Methods

The current animal model‐based randomised controlled trial was conducted from January 2022 to June 2023 at the Research and Experimental Centre of Gansu University of Chinese Medicine.

### 2.1 Animals and Groups

Male Sprague–Dawley pathogen‐free rats (20 months old, weighing 300–400 g) were used in all the experiments. The experimental rats were purchased from the Experimental Animal Centre of Gansu University of Traditional Chinese Medicine (Licence No.: SCXK [Gan] 2020‐0001) and kept under standardised conditions, with free access to water and food. Seventy‐two rats were randomly divided into four groups: control, surgery, EA and resveratrol. Each group was divided into two subgroups according to the different time points after surgery, a 3‐day postoperative group and a 7‐day postoperative group, with eight animals in each group (*n* = 8). Rats in the surgical group were subjected to laparotomy, and postoperative behavioural tests were performed, after which the rats were sacrificed for extraction. Rats that did not receive any treatment were used as controls. The resveratrol group received an intraperitoneal injection of resveratrol, a selective SIRT1 agonist, once a day at 10 mg/kg, seven times before surgery. This study was approved by the Ethics Committee of Gansu University of Traditional Chinese Medicine (Animal Ethics Approval No. 2021‐200). Animal handling followed the Guidelines for the Kind Treatment of Laboratory Animals.

### 2.2 Surgery and Anaesthesia

The rats were anaesthetised by intraperitoneal administration of propofol (150 mg/kg), a 3 cm incision was made in the abdomen to stimulate the abdominal viscera and muscles and anaesthesia was maintained for 2 h. The rats were fixed to expose their abdomens, and the skin and muscles were gradually separated along the mid‐abdominal line. The abdominal cavity was fully exposed; the rats' small intestine, caecum, and large intestine were gradually explored with forceps; and the small intestine was rubbed with gauze for 3 min three times at 7 min each time interval. The wounds were sutured layer by layer and sterilised with iodophor. The entire surgical time was approximately 30 min. After recovery, the rats were returned to their cages for warmth, maintained individually and kept individually after returning to their cages. After surgery, 1% povidone‐iodine was applied to the rat wounds twice daily until the wounds healed.

### 2.3 Sample Collection and Conservation

After completing the behavioural test, blood was collected through the abdominal aorta. The plasma was allowed to stand for approximately 30 min at room temperature and centrifuged in a low‐temperature centrifuge (3000 rpm) for 15 min, and then the serum cryopreserved tubes were labelled and dispensed and placed in an ultra‐low‐temperature refrigerator at −80°C for storage. After blood collection, the rats were sacrificed and the scalp was cut off with tissue scissors along the median sagittal line to expose the skull. The parietal bones were separated using surgical scissors to expose the brain tissue, which was then gently and completely removed. The brain tissue was placed on an ice plate and carefully separated layer by layer with curved forceps to fully expose and completely remove the bilateral hippocampal tissue. The hippocampal tissues for reactive oxygen species (ROS) detection were placed in Hank's balanced salt solution after removal, and the remaining hippocampal tissues were placed in freezing tubes and stored in an ultra‐low temperature refrigerator at −80°C for spare parts.

### 2.4 EA

In the EA group, the selected acupoints were Baihui (GV20, right in the middle of the parietal bone) and Neiguan (PC6, inner forelimb, between the radial‐ulnar suture approximately 3 mm from the carpal joint), and EA was performed based on Sun et al. [31] EA pre‐treatment was performed 5 days before surgery, and EA was performed simultaneously every day. All the needles were connected to the end of the HANS acupoint neurostimulator (HANS‐LH202H), and the stainless steel needles (0.16 mm × 7 mm) were vertically inserted into the GV20 and the PC6 at a depth of 2–3 mm from the vertical angle. The EA parameters were set as follows: 2/100 Hz sparse‐dense wave with an intensity of 1 mA for 30 min, once a day for five consecutive days.

### 2.5 Morris Water Maze (MWM)

The rats' cognitive and memory functions were assessed using the MWM experiment. 1 week before surgery, the rats were tested for five consecutive training days, scheduled four times daily for a fixed period. During training, the platforms were placed in the same quadrant, and each mouse was released into the water from any starting point on the four pool walls (facing walls) and was allowed to locate the hidden platform. The average of the four training latencies of the rats was considered the learning performance of the rats on that day. A spatial exploration test was performed 3 and 7 days after surgery. The platforms were removed, and the rats were placed in water to swim. The swimming paths of the rats were recorded within 120 s, and the residence time in the target quadrant and the number of times the rats crossed the target quadrant were recorded.

### 2.6 Quantitative Real‐Time Polymerase Chain Reaction (qPCR)

RNA was extracted from the rat hippocampal tissue using a Tissue Total RNA Extraction Kit. qPCR analysis was performed using the Hifair III 1st Strand cDNA Synthesis SuperMix qPCR kit (Yeasen Biotechnology). qPCR analysis was performed on a LightCycler 96 System using the Hieff qPCR SYBR Green Master Mix kit. qPCR analysis was performed at 95°C for 5 min, followed by 40 cycles of 95°C for 10 s and 60°C for 30 s. The qPCR analysis was performed on a LightCycler 96 System using the Hieff qPCR SYBR Green Master Mix Kit (Yeasen Biotechnology). GAPDH‐normalised PCR results and gene expression levels were measured by the $2^{-\Delta \Delta C_{t}}$ method. The primer sequences are listed in Table 1.

**TABLE 1:** Sequences of rat‐specific primers used for RT‐PCR: SIRT1 and FOXO1.

Gene	Primer sequences
SIRT1	F: TAATCAGGTAGTTCCTCGGTG
	R: TCGCAGTCTCCAAGAAGC
FOXO1	F: AAGCATCTCTCCAGTCCG
	R: AGTGTAGACGCCATCTTGG
GAPDH	F: CATCTTCTTGTGCAGTGCC
	R: ACCAGCTTCCCATTCTCAG

### 2.7 Protein Blotting

Hippocampal tissue was dissolved in radio immunoprecipitation assay lysis buffer (Solarbio), and the total protein concentration was quantified using a BCA Protein Assay Kit (Solarbio) according to the manufacturer's instructions. A total of 20 μg of protein was loaded into lanes of an 8% SDS PAGE gel, electrophoresed and then transferred to a PVDF membrane (Millipore). The membranes were then closed with 10% skimmed milk for 1.5 h at room temperature and then incubated at 4°C with sirt1 (1:1000, ab32536, Abcam), FOXO1 (1:1000, YT1757, Immunoway), Beclin1 (1:1000, ab207612‐40, Abcam), P62 (1:1000, ab109012, Abcam) and other primary antibodies, which were incubated overnight. The membrane was then washed and incubated with the corresponding secondary antibody (1:10,000, ab6721; Abcam). The membranes were then incubated with a chemiluminescent substrate (NCM Biotech), and the images were exposed and processed using an exposure machine (BLT Photon Technology) and ImageJ software.

### 2.8 Flow Cytometry

Tissue was sheared in Hanks salt solution and added to the enzyme lysate, followed by 10 μL of DNA enzyme digested at 37°C for 15–30 min. The digested tissue solution was homogenised using a pipette and filtered through a 300 mesh sieve, centrifuged at 300 *g* for 5–10 min, resuspended in 5 mL of 30% personal and centrifuged at 300 *g* for 10 min to get the precipitate, Hbss was resuspended and centrifuged, and the supernatant was discarded. The precipitate was resuspended in a 0.4 μL DCFH (1:1000 dilution, Dalian Meilun Biotechnology MA0219) probe and incubated for 15–30 min away from light. The data were obtained and analysed using a MACSQuant flow cytometer.

### 2.9 Enzyme‐Linked Immunosorbent Assay (ELISA)

Rat blood was collected, coagulated at room temperature for 10–20 min and centrifuged for approximately 20 min (2000–3000 r/min), and the supernatant was collected for ELISA. Relative levels of superoxide dismutase (SOD, YJ059387, Shanghai Enzyme‐linked Biotechnology) and catalase (CAT, YJ037079, Shanghai Enzyme‐linked Biotechnology), malondialdehyde (MDA item number Shanghai Enzyme‐linked Biotechnology), and S100 calcium‐binding protein B (S100‐B, YJ028440, Shanghai Enzyme‐linked Biotechnology) in rat serum were determined according to the manufacturer's protocol.

### 2.10 Statistical Analysis

All data were analysed using GraphPad Prism 9.3.0 (GraphPad Software) statistical software. Quantitative data were expressed as mean ± standard deviation (SD). Data from the MWM test were analysed using a two‐way analysis of variance (ANOVA) with repeated measures. Other data were analysed using a one‐way ANOVA and Tukey's test. In this study, all two‐sided tests were used to compare whether the differences between groups were statistically significant or not. *p* < 0.05 was statistically significant.

## 3 Results

### 3.1 EA Treatment Improves Cognitive Performance in POCD Rats

**FIGURE 1:**
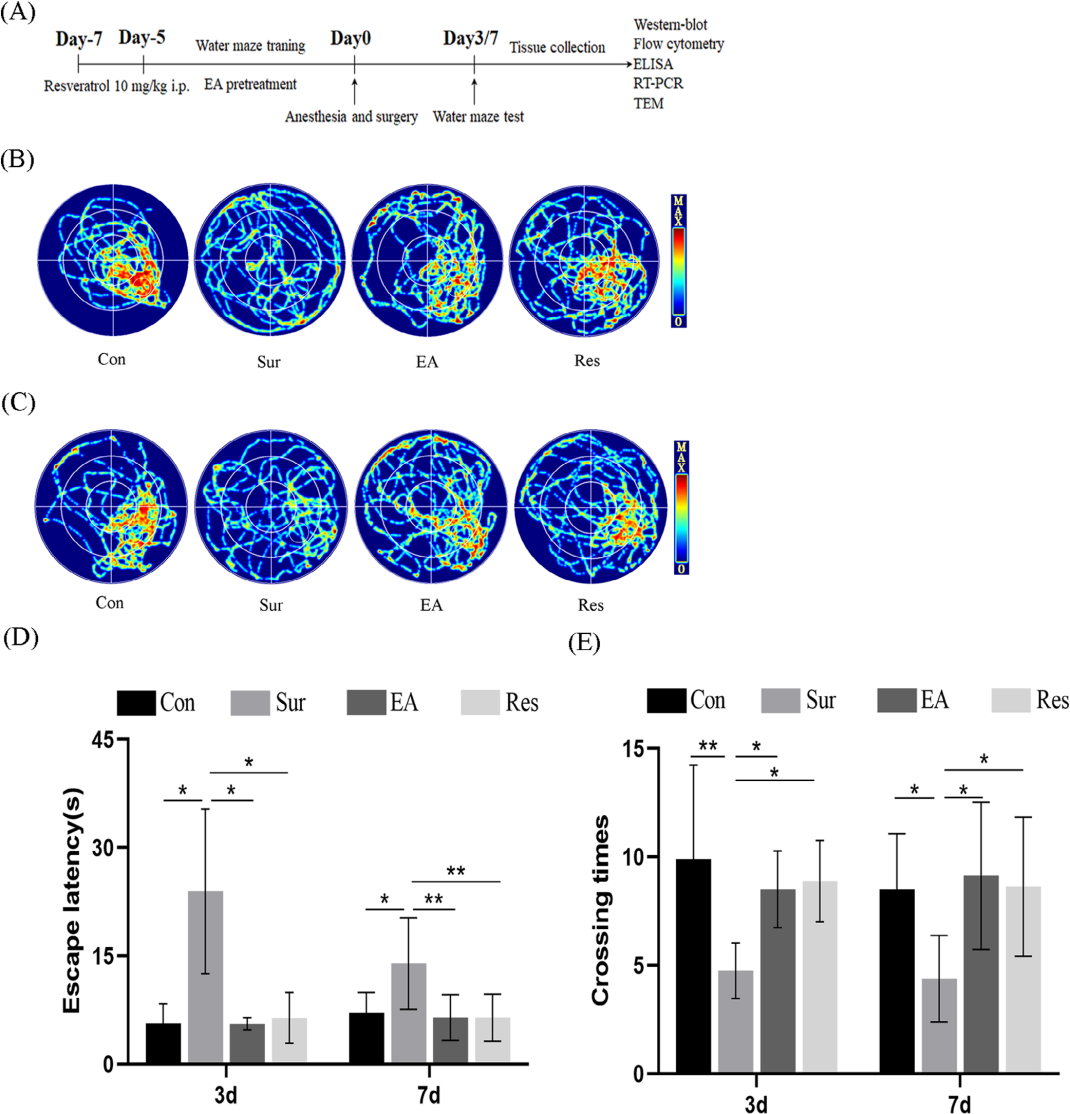
EA treatment improved cognitive function in aged POCD rats. (A) The schematic diagram illustrating the chronological events of experiments. (B, C) The number of platform traversals at 3 and 7 days postoperatively was analysed by MWM. (D) A hidden platform training test is used to assess memory, and an (E) spatial exploration experiment is used to assess learning. Each data point is mean ± SD (*n* = 8). *p < 0.05, **p < 0.01.

The MWM test was performed to test whether EA treatment could improve the learning and memory abilities of POCD rats (Figure 1A). As shown in Figure 1C,D, the experimental results showed that the postoperative escape latency was significantly prolonged, and the number of platform traversals was significantly reduced in the surgical group of rats compared to the control group, indicating that old rats experiencing anaesthesia/surgery showed a decline in spatial memory and cognitive ability.

In the hidden platform test, the escape latency of rats in the surgical group was prolonged compared to that of control rats during anaesthesia and on days 3 and 7 after surgery. Pre‐surgical EA pre‐treatment significantly shortened the escape latency, indicating that EA improved memory in aged POCD rats (Figure 1D). In the spatial exploration test, the number of platform traversals was significantly reduced in the surgical group of rats compared to that in the control group. In contrast, EA pre‐treatment reversed these changes (Figure 1B,C,E), suggesting that EA treatment improved the learning ability of aged POCD rats.

### 3.2 EA Pre‐Treatment Attenuates Postoperative Oxidative Stress in Aged Rats

**FIGURE 2:**
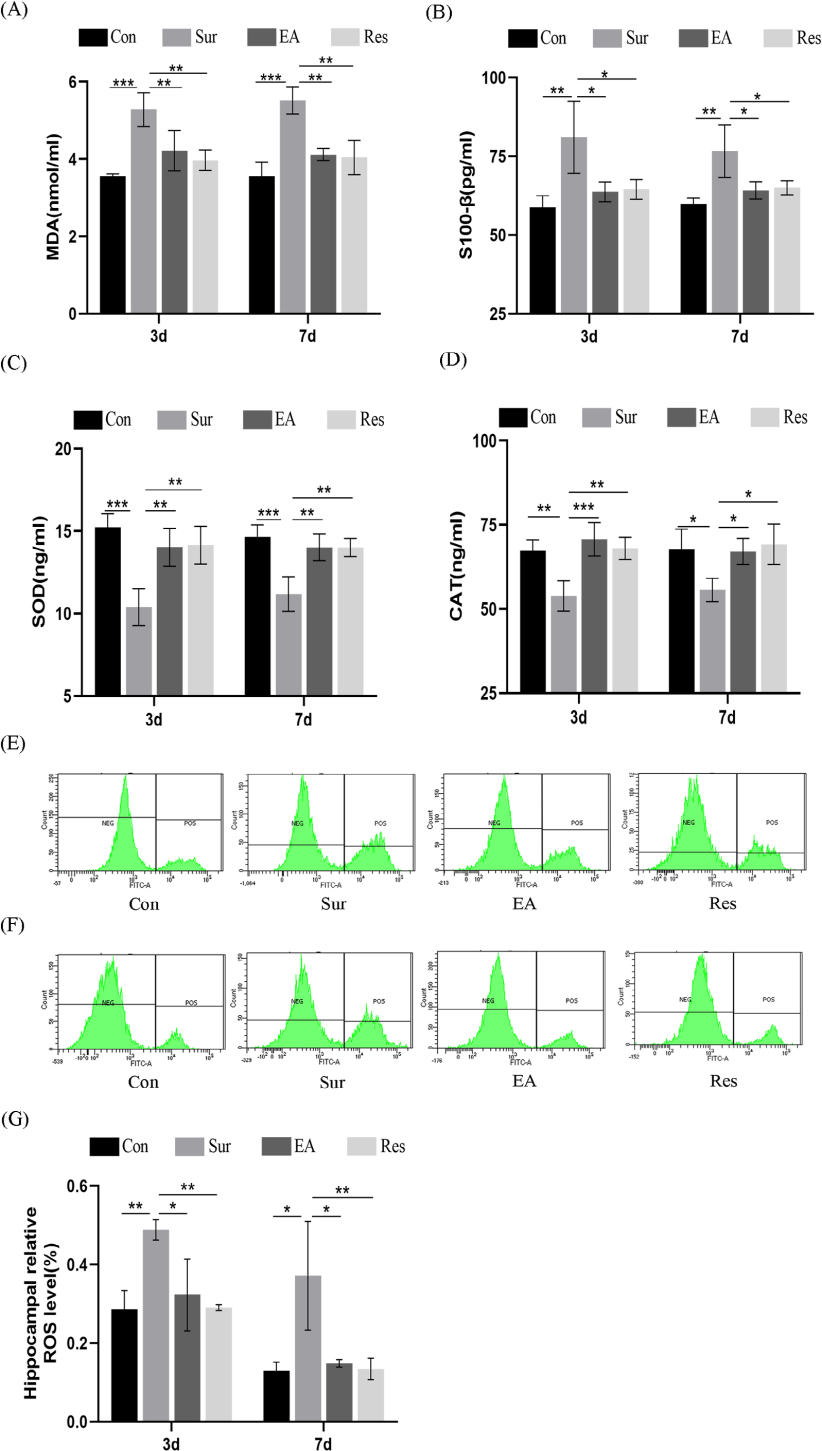
EA pre‐treatment attenuates oxidative stress after surgery in aged rats. (A) MDA levels, (B) S100B levels, (C) SOD levels and (D) CAT levels in serum were evaluated by the indicated ELISA kits in each group of rats at 3 and 7 days (*n* = 6); (E–G) Flow cytometry was used to detect ROS activity in the hippocampus of each group of rats at 3 and 7 days after surgery (*n* = 3). Data are represented as mean ± SD. *p < 0.05, **p < 0.01, ***p < 0.001.

Surgical trauma‐associated oxidative stress is a key pathogenic factor in POCD. The concentration of MDA in the serum of the rats in the surgery group was significantly higher than that in the control group (Figure 2A). The changes in the level of S100‐β (Figure 2B) in all groups behaved similarly to changes in MDA levels, and EA pre‐treatment reversed these changes. In contrast, as shown in Figure 2C,D, the serum levels of SOD and CAT were reduced in aged POCD rats, while EA treatment significantly increased the levels of SOD and CAT in the hippocampus and reduced the levels of ROS and MDA. Significant differences were observed in the changes in ROS concentrations among the groups. As shown in Figure 2E,F, flow cytometry was performed to detect the activity of ROS in the hippocampus of the day 3 and day 7 postoperative groups, respectively. The results indicated that POCD increased the expression of ROS in the hippocampus and restored the upregulation of ROS by EA pre‐treatment (Figure 2G), indicating that EA pre‐treatment suppressed oxidative stress induced by POCD. These data suggest that oxidative stress is activated in the hippocampus of aged rats and that EA pre‐treatment suppressed oxidative stress.

### 3.3 EA Pre‐Treatment Activates the SIRT1/FOXO1 Pathway

**FIGURE 3:**
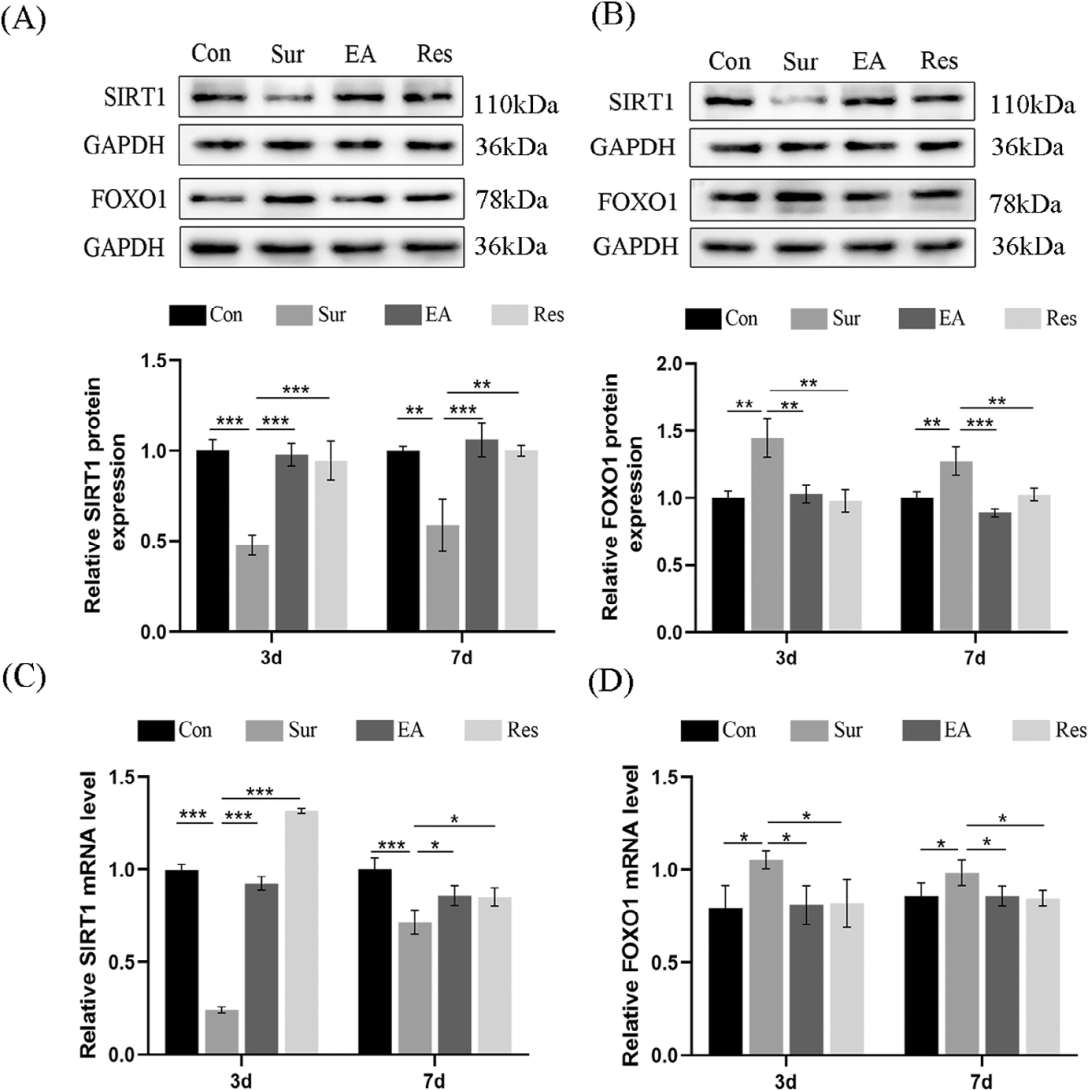
EA pre‐treatment activates the Sirt1/FOXO1 pathway. (A, B) Protein blotting and semi‐quantification of SIRT1 and FOXO1 protein levels in hippocampal tissues on postoperative days 3 and 7. (C, D) qPCR analysis of SIRT1 and FOXO1 mRNA levels in hippocampal tissues. Each data point is mean ± SD (*n* = 5). *p < 0.05, **p < 0.01, ***p < 0.001.

To determine whether EA modulates the Sirt1/FOXO1 pathway, we examined protein and mRNA levels associated with this pathway. In the current study, we observed that the agonist group upregulated Sirt1 expression, while hippocampal FOXO1 expression was significantly decreased. Similarly, EA treatment significantly increased Sirt1 protein expression and decreased FOXO1 protein levels in the hippocampus of POCD rats (Figure 3A,B). In addition, SIRT1 mRNA levels were significantly downregulated in the hippocampi of POCD rats (Figure 3C), and FOXO1 mRNA expression levels were significantly upregulated (Figure 3D). These results showed that preoperative EA pre‐treatment upregulated the expression of SIRT1, activated the SIRT1/FOXO1 pathway and reversed anaesthesia/surgery‐induced cognitive deficits.

### 3.4 EA Improves POCD by Activating Autophagy Through the SIRT1/FOXO1 Pathway

**FIGURE 4:**
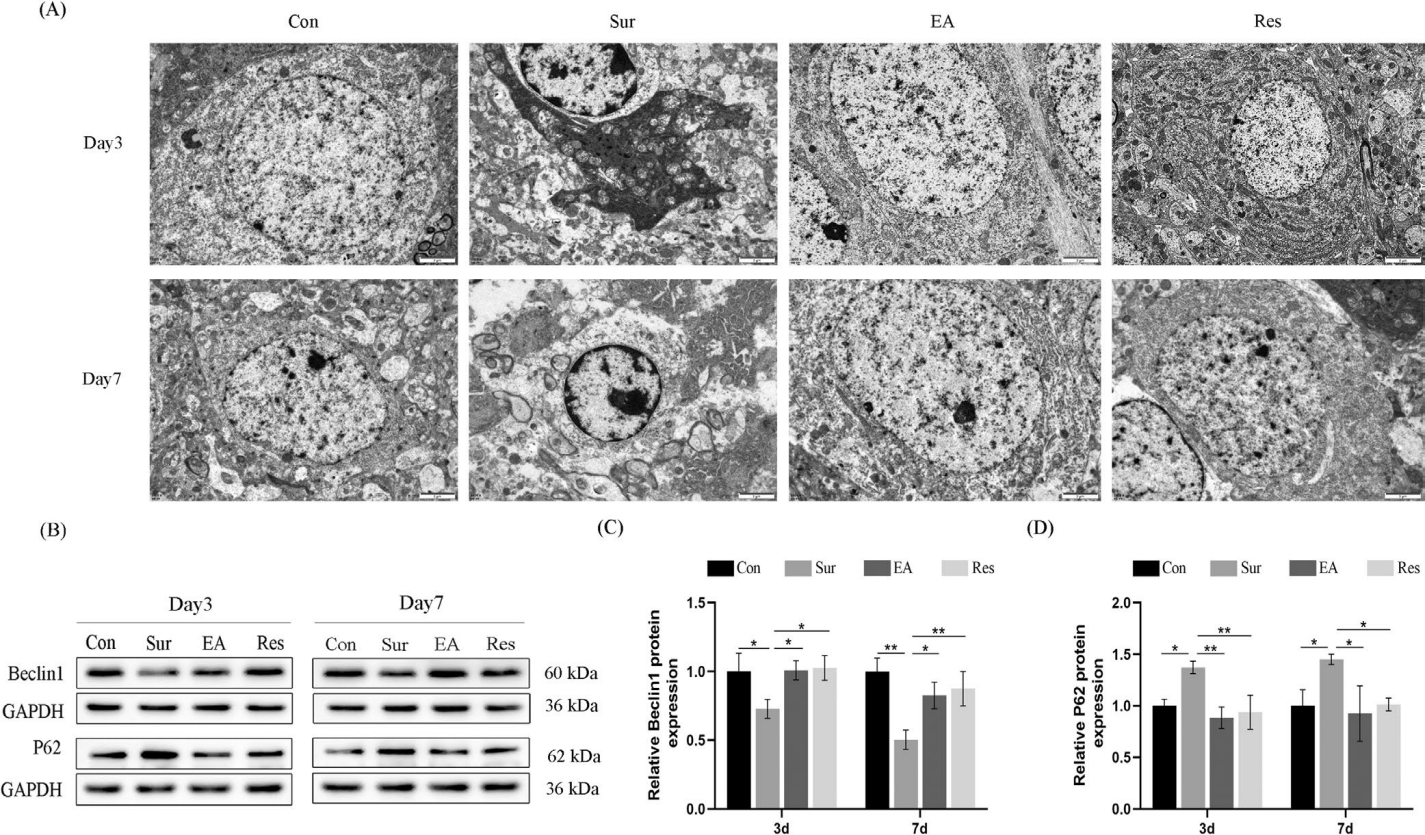
EA improves POCD by activating autophagy through the SIRT1/FOXO1 pathway. (A) Representative neuronal ultra microstructure in different groups, scale bar = 2 μm. (B–D) Protein blotting and semi‐quantification of Beclin 1, P62 protein levels in hippocampal tissues on postoperative days 3 and 7. Each data point is mean ± SD (*n* = 5). *p < 0.05, **p < 0.01.

We used transmission electron microscopy (TEM) to observe the hippocampal ultrastructure in different groups. The results showed that in the control group, the cell nuclei had regular morphology, with smooth surfaces, clear double nuclear membranes, normal nuclear perinuclear space and no agglutination of chromatin in the nucleus. The number of mitochondria in the cytoplasm was abundant, and a few were oedematous. Compared with the control group, the neuronal cells in the surgical group were swollen and necrotic, and the cells were solidified and deep‐stained. The nuclei had irregular morphology, with uneven surfaces and an unclear structure of the double nuclear membranes, and a large number of mitochondria in the cytoplasm were oedematous. The cristae were reduced or disappeared. Morphology of mitochondria was observed to show structural atrophy, with some mitochondria showed deletion; some mitochondrial autophagy was observed, and neuronal apoptosis induced by mitochondrial disruption significantly increased. Pre‐treatment could reverse this phenomenon in the surgical group (Figure 4A).

Autophagy is regulated by many signalling pathways, among which the Sirt1/FOXO1 pathway is a major pathway that regulates autophagy in endothelial cells [25, 34]. We examined the expression levels of autophagy‐related proteins Beclin‐1 and P62 in aged POCD rats. As shown in Figure 4B, in POCD rats, we observed a decrease in the expression level of Beclin‐1 and a significant increase in the level of P62 protein. In contrast, EA treatment suppressed the increased expression of P62 and upregulated Beclin‐1 expression. When the SIRT1/FOXO1 signalling pathway is inhibited, autophagy is downregulated. In addition, EA treatment increased the expression of Beclin1, decreased the expression of p62 through activating the SIRT1/FOXO1 pathway (Figure 4C,D) and increased autophagy in aged rats. The above results indicated that EA pre‐treatment upregulated the expression of SIRT1, activated the SIRT1/FOXO1 pathway and reversed the cognitive deficits caused by anaesthesia/surgery‐induced inhibition of autophagy.

## 4 Discussion

EA pre‐treatment demonstrates improved postoperative cognitive dysfunction by inducing SIRT1 expression and activating the SIRT1–FOXO1 signalling pathway and oxidative stress. POCD is common in older adult patients and is a common complication of anaesthesia and surgery. Oxidative stress damage increases with age as senescent cells and damaged proteins accumulate. Autophagy can process and degrade these toxic products to protect hippocampal neurones from oxidative stress damage. However, the protective effects of autophagy are weakened by ageing, drugs and injury, resulting in impaired neurocognitive function. This study aimed to explore the neuroprotective effects and potential mechanisms in terms of hippocampal autophagy and oxidative stress by EA pre‐treatment to ameliorate surgical/anaesthesia‐induced cognitive deficits in adult rats. These findings suggest that EA‐induced sirtuin activation may be involved in ameliorating autophagy‐related postoperative cognitive deficits. In the current study, we observed that EA treatment ameliorated POCD induced by propofol anaesthesia or caesarean section in aged rats. EA‐induced overexpression of sirt1 protects cognitive function in rats with POCD. The SIRT1/FOXO1 autophagy signalling pathway and oxidative stress may participate in this process.

Sirt1 plays an essential role in hippocampal neuronal development and protects neurones from injury and apoptosis [35]. As reported by Ma et al. [36] the reduction in Sirt1 due to repeated propofol exposure can mediate synaptic plasticity and neuronal excitability impairments, which may cause a decline in learning and memory. In contrast, Sirt1 activation prevents microglia‐mediated synaptic phagocytosis after anaesthesia and surgery and reverses cognitive deficits [37]. As a major downstream target of Sirt1, FOXO1 has been confirmed to be regulated by SIRT1 deacetylation, which plays a key role in cellular stress adaptation and directly regulates the transcription of autophagy‐related genes by binding to the promoter region [38]. SIRT1 reduces diabetes‐induced renal injury by synergistically working together through FOXO1‐mediated oxidative stress and autophagy [39]. Sirt1 overexpression activates the Sirt1/FOXO1 signalling pathway, which enhances resistance to oxidative stress injury, ameliorates bone loss, and promotes osteogenesis [40].

Resveratrol is a natural compound that activates SIRT1 [41], which can regulate acetylated tau and hyperphosphorylated tau levels in the hippocampus and inhibit hippocampal neuroinflammation and antioxidant and anti‐apoptotic effects. Resveratrol activates the SIRT1‐related pathway effectively in various diseases and plays an essential role in brain neuroprotection. Numerous studies have shown that resveratrol can activate SIRT activity and upregulate SIRT expression to exert anti‐ageing, anti‐oxidative stress and inflammatory effects [42, 43, 44]. Resveratrol improves postoperative cognitive dysfunction by activating the SIRT1 pathway and upregulating autophagy levels [45]. Studies have shown that resveratrol can enhance the body's resistance to oxidative damage by activating the SIRT1/FOXO1 signalling pathway [46]. Activation of SIRT1 levels in RES‐induced diabetic mice promotes the deacetylation of FOXO1, leading to the activation of autophagy by binding to the Rab7 promoter [47]. Resveratrol exerts cardioprotective effects by activating the SIRT1/FOXO1 pathway, reducing oxidative stress and inducing cellular autophagy [48].

In the current study, the SIRT1 agonist resveratrol was given pre‐treatment before surgery. Western blot detected the protein expression in hippocampal tissues, and the changes in SIRT1 and FOXO1 mRNA levels were detected using qPCR. The results showed that by administering resveratrol to the rats in the Res group, the expression of SIRT1 protein was increased after anaesthesia and surgery. In contrast, the level of FOXO1 protein was decreased, and the same expression was observed at the mRNA level. These results suggest that anaesthesia surgery‐induced downregulation of SIRT1 expression and elevation of FOXO1 expression in hippocampal tissues of aged rats, decrease in the SIRT1 mRNA level, and elevation of FOXO1 mRNA are closely related to the downregulation of the SIRT1/FOXO1 pathway, which may activate the SIRT1/FOXO1 pathway by upregulating the expression of SIRT1 and improving cognitive ability.

Beclin‐1 is an Atg6/VPS30 direct homologue with an essential role in autophagic processes and protein sorting [49]; its function depends on the interactions between autophagy‐related genes of the autophagy pathway and regulatory proteins of the apoptosis pathway [50]. Beclin‐1 is a commonly used indicator of autophagy levels and is an important factor in regulating autophagy dysfunction‐related diseases. Studies have shown that in Alzheimer's disease, Beclin1‐mediated autophagy exerts a cytoprotective effect mainly by regulating amyloid precursor protein processing. When Beclin1 levels are decreased, autophagy levels also decrease, leading to the accumulation of protein aggregates, the most common pathological feature of Alzheimer's disease [51, 52]. p62/SQSTM1 is a key selective autophagy receptor whose oligomerisation is essential for phagocytosis to correctly target ubiquitinated substrates [9] and plays a vital role in cell metabolism, signalling and apoptosis [53]. The inhibition of autophagy or disruption of protease homeostasis results in the accumulation of p62, which can be used in response to autophagic flux [54, 55]. AMPKα1 overexpression activates autophagic signalling by increasing Beclin1 expression and decreasing p62 expression in the hippocampus of POCD rats, which improves postoperative cognitive function [56].

The SIRT1/FOXO1 signalling pathway is involved in the regulation of a variety of physiological processes, and SIRT1 can play a synergistic role in attenuating diabetes‐induced renal injury through FOXO1‐mediated oxidative stress and autophagy together [39, 57]. In addition, miR‐181a upregulation induced by oxidative stress mediated the downregulation of SIRT1 and increased the level of FOXO1 acetylation, which caused the aggregation of FOXO1 in the nucleus and induced apoptosis in granulosa cells [58]. Melatonin exerted antioxidant and anti‐inflammatory effects through activating SIRT1 and the deacetylation of FOXO1 and p65 [59]. Hariharan et al. [60] reported that SIRT1 mediates FOXO1 deacetylation to induce autophagy by inducing the small GTPase Rab 7 expression. Studies have shown that resveratrol prevents arterial thrombosis by enhancing autophagic flux by activating the Sirt1/FOXO1 pathway [38]. In addition, SIRT1/FOXO1 signalling may be involved in cognitive impairment by regulating autophagy [61].

Autophagy [14] and oxidative stress [62] play key roles in POCD development. Autophagy is a key mechanism that protects neurones from anaesthetic surgery‐induced apoptosis. When hippocampal autophagy levels are suppressed, abnormal aggregation of α‐synuclein oligomers and altered neurotransmitter levels in the hippocampus of aged rats after propofol anaesthesia and caesarean section ultimately lead to cognitive deficits [63]. Similarly, in the current study, anaesthesia and surgery caused a significant decrease in Beclin1 protein levels and a significant increase in p62 protein levels in the rat hippocampus, suggesting that the development of POCD in rats is associated with the downregulation of hippocampal autophagy levels.

Accumulation of ROS and oxidative damage are characteristic features of senescent cells. ROS accumulation disrupts the integrity of the mitochondrial membrane, and oxidative stress is one of the key factors leading to cognitive deficits in the postoperative period. Inhibition of oxidative stress ameliorates anaesthesia/surgery‐induced cognitive decline [64]. In our study, anaesthesia/surgery disrupted the structural integrity of mitochondria and the normal morphology of cells, increased the levels of ROS and MDA in hippocampal tissues and was accompanied by a decline in neurocognitive performance, suggesting a relationship between oxidative stress and cognitive deficits in the postoperative period in rats.

EA stimulation has been recognised as a viable strategy for improving learning and memory abilities in POCD [32, 65]. However, the potential mechanisms underlying the cognitive improvement after EA treatment remain unclear. Previous studies have shown that EA treatment promotes the activation of hippocampal mitochondrial autophagy and ameliorates cognitive deficits in POCD rats [66]. Therefore, we hypothesised that EA pre‐treatment could improve cognitive dysfunction induced by propofol anaesthesia or dissection and that mitochondrial autophagy and oxidative stress might be related. To further assess the necessity of activation of autophagy and inhibition of oxidative stress in the therapeutic effect of EA, Sirt agonist application showed that activation of the SIRT1/FOXO1 pathway markedly improved cognitive ability in aged rats; as evasive latency was shortened, the number of plateau traversals was increased, autophagy‐associated protein levels were upregulated and oxidative stress was inhibited. As expected, EA treatment induced these changes, confirming that EA plays a role in upregulating autophagy and attenuating oxidative stress through the SIRT1/FOXO1 pathway. In the current study, our findings demonstrated that EA pre‐treatment improves anaesthesia/surgery‐induced hippocampus‐dependent learning and memory. EA‐induced Sirt1 overexpression promoted hippocampal mitochondrial autophagy activation and inhibited hippocampal oxidative stress. Our study suggests that EA‐induced activation of the SIRT1/FOXO1 autophagy signalling pathway and oxidative stress are involved in ameliorating postoperative cognitive impairment.

There are limitations to the current study. It remains to be further explored whether the activation of the SIRT1/FOXO1 signalling pathway by EA stimulation interacts with other related pathways and thus has a role in the inhibition of oxidative stress and the activation of autophagy. Moreover, whether oxidative stress and autophagy act in parallel or cascade is not clear, and the relationship between the two needs to be further explored.

In conclusion, we demonstrated in the current study that EA alleviates cognitive dysfunction and ameliorates autophagy inhibition and oxidative stress in an aged POCD rat model. The therapeutic effects of EA depend on the activation of autophagy and inhibition of oxidative stress. Therefore, EA may prevent learning and memory deficits in POCD rats by activating the SIRT1/FOXO1 signalling pathway and autophagy.

## Author Contributions

**Xiaojia Zhang:** data curation (equal), software (equal), writing – original draft (equal), writing – review and editing (equal). **Binsen Zhang:** data curation (equal), investigation (equal). **Xiaoyu Qin:** conceptualization (equal), methodology (equal), resources (equal). **Lu Tang:** investigation (equal), software (equal). **Chunai Wang:** funding acquisition (equal), methodology (equal), resources (equal).

## Conflicts of Interest

The authors declare no conflicts of interest.

## Data Availability

The data that support the findings of this study are available from the corresponding author upon reasonable request.
